# Characterization and Identification of a woody lesion mimic mutant *lmd*, showing defence response and resistance to *Alternaria alternate* in birch

**DOI:** 10.1038/s41598-017-11748-2

**Published:** 2017-09-12

**Authors:** Ranhong Li, Su Chen, Guifeng Liu, Rui Han, Jing Jiang

**Affiliations:** 1 State Key Laboratory of Tree Genetics and Breeding, Northeast Forestry University, Harbin, 150040 China; 2 Department of Life Science & Technology, Mudanjiang Normal University, Mudanjiang, 157100 China

## Abstract

Lesion mimic mutants (LMM) usually show spontaneous cell death and enhanced defence responses similar to hypersensitive response (HR) in plants. Many LMM have been reported in rice, wheat, maize, barley, *Arabidopsis*, etc., but little was reported in xylophyta. BpGH3.5 is an early auxin-response factor which regulates root elongation in birch. Here, we found a T-DNA insertion mutant in a *BpGH3*.5 transgenic line named *lmd* showing typical LMM characters and early leaf senescence in *Betula platyphylla* × *B*. *pendula*. *lmd* showed H_2_O_2_ accumulation, increased SA level and enhanced resistance to *Alternaria alternate*, compared with oe21 (another *BpGH3*.5 transgenic line) and NT (non-transgenic line). Cellular structure observation showed that programmed cell death occurred in *lmd* leaves. Stereomicroscope observation and Evans’ blue staining indicated that *lmd* is a member of initiation class of LMM. Transcriptome analysis indicated that defence response-related pathways were enriched. Southern-blot indicated that there were two insertion sites in *lmd* genome. Genome re-sequencing and thermal asymmetric interlaced PCR (TAIL-PCR) confirmed the two insertion sites, one of which is a T-DNA insertion in the promoter of *BpEIL1* that may account for the lesion mimic phenotype. This study will benefit future research on programmed cell death, HR and disease resistance in woody plants.

## Introduction

Plants experience spontaneous cell death during development and as a means of resistance to harmful environments, which is called programmed cell death (PCD)^1^. PCD influences the whole cyclogeny of plant, including germination, vegetative and reproductive growth, senescence, and especially the response to biotic or abiotic stress^2^. When attacked by imcompatible pathogens, plants can initiate a form of partial PCD at the infected sites to limit the pathogen spread. This is an innate immune response called hypersensitive response (HR). At the same time, certain defence-related genes are activated and systemic acquired resistance (SAR) is formed to protect the plants from attacking^3^.

Lesion mimic mutants (LMM) are a class of mutants that show spontaneous cell death and defence response without any pathogen attack^4^. The lesions of LMM resulting from the altered regulation of cell death processes resemble to HR-mediated cell death. LMM have been ideal materials to study cell death and defence pathways in plants. Currently, many LMM have been isolated from *Arabidopsis* ^5^, rice^6^, maize^7^, barley^8^, wheat^9^, potato^4^, and cotton^10^, etc. The genes that have been cloned in LMM encode various proteins involved in different pathways including chloroplast activity and light energy, sphingolipids and fatty acids metabolism, signal perception, ion fluxes and reactive oxygen species (ROS) changes^11^, indicating a complex mechanism of LMM formation. The overexpression of certain genes can also lead to the lesion mimic phenotype^12, 13^. LMM are usually divided into two groups: the initiation class, which shows location lesions and is discrete in size, and the propagation class, which includes runaway cell death wherein lesions, once formed, can expand continuously to the entire tissue^14^. Most LMM are accompanied by molecular and cellular changes such as defence-related genes expression, callose deposition, ROS accumulation, increased salicylic acid level and activation of SAR^15^. Some important phytohormones, including salicylic acid (SA), jasmonic acid (JA) and ethylene, are usually involved in a complex pathogen-plant interaction^16–18^. These phytohormones also play important roles in different LMM.

Reactive oxygen species (ROS), such as superoxide anion radical and hydrogen peroxide (H_2_O_2_), are important factors that signal the onset of abiotic and biotic stress. Different damaging and protective signalling pathways are activated by ROS. Many transcription factors (AS1, MYB30, MYC2 and WRKY70), hormone regulators (AXR1, ERA1, SID2, EDS1 and SGT1b) and cell death regulators (RCD1 and DND1) are regulated in H_2_O_2_-mediated cell death^19^.

Plants have evolved sophisticated defence systems to protect themselves from various biotic and abiotic attacks^20^. Once attacked by a pathogen, the plant immune system is triggered immediately by pathogen-associated molecular patterns (PAMPs) which is called PAMP-triggered immunity (PTI). PTI constitutes a basic defence response^21^. Effector recognition is mediated by R protein. The R protein can activate effector-triggered immunity (ETI) during the interaction between the pathogen and plants after PTI. Most R proteins contains nucleotide binding site (NBS) and conserved leucine-rich-repeat (LRR) domain^22^. ETI induces SA accumulation and MAPK activation, which are important for plant disease resistance^23^. This is followed by activation of the SAR. SA plays a critical role in this process and induces the expression of pathogenesis-related genes (*PR1*, *PR2*, *PR*5)^24^. Furthermore, many plants including tomato, wheat, and *Arabidopsis* ^25–27^, etc., show increased SA level after pathogen infection. The most direct evidence of the importance of SA function is provided by transgenic tobacco plants expressing the NahG gene. NahG gene encodes salicylate hydroxylase which can convert SA to catechol. *NahG* overexpression in tobacco can inhibit SA accumulation and the transgenic line defective in the ability to induce SAR against tobacco mosaic virus^28^. Besides, the PeaT1-induced SAR pathway is also mediated by salicylic acid and NPR1 gene^29^.

Ethylene (ET) is an endogenous hormone involved in seed germination, organ abscission, fruit ripening, senescence, disease and stress resistance^5^. ET and SA usually act synergistically to confer disease resistance in plants. ET treatments on *NahG* transgenic tobacco can elevate SA level. Ethylene insensitive 3 (EIN3) which belongs to the EIN3 family is a positive regulator of the ET signal transduction^30^. The degradation of EIN3 protein is a primary means by which the sensitivity of plants to ethylene is regulated^31^. There are five EIN3-like proteins (EILs), namely, EIL1, EIL2, EIL3, EIL4, EIL5, among which EIL1 is the most similar and functionally redundant to EIN3 in *Arabidopsis thaliana*. EIN3/EIL1 can activate many genes related to ethylene response^32^, senescence^33^, flower development^30^, SA production^34^, salt stress^35^, pathogen invasion^36^, iron homeostasis^37^, and Fe metabolism^38^, indicates that EIN3/EIL1 is a junction of different pathways.

In this study, we described a T-DNA insertion mutant of *Betula platyphylla* × *B*. *pendula* named *lmd* with a typical LMM phenotype. The *lmd* mutant exhibited spontaneous cell death and leaf abscission both *in vitro* and in soil. Southern blotting showed that there were two insertion sites in the *lmd* genome. Genome re-sequencing and TAIL-PCR results indicated that the insertion of *BpEIL1* promoter might cause the formation of the lesion mimic phenotype. The lesions formation and the enhanced defence response are related to SA accumulation. Our study characterized and identified a woody lesion mimic mutant in birch. It suggests that *BpEIL1* plays a crosstalk role in the signalling cascade leading to defence response and HR cell death.

## Results

### Characterization of *lmd* mutant

**Figure 1:**
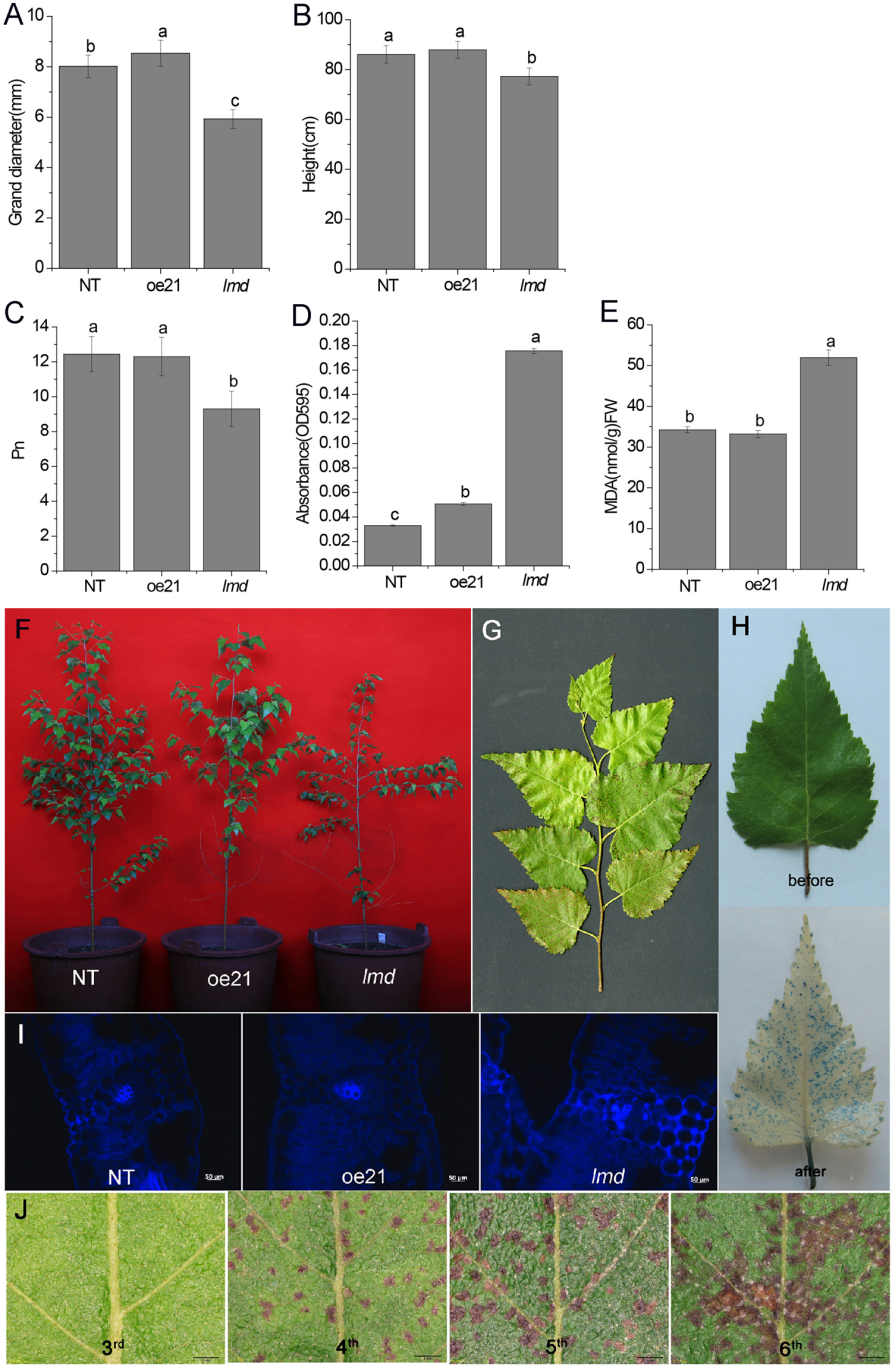
Phenotype of the *lmd* mutant. (**A** and **B**) Show the ground diameter and height, respectively. (**C**) Net photosynthetic efficient (Pn). (**D**) Absorbance of NT, oe21 and *lmd* eluent. The absorbance of *lmd* is higher than that of NT and oe21which indicated that the cell death is more in *lmd* than in NT and oe21. (**E**) Comparison of MDA content in NT, oe21 and *lmd*. (**F**) Increment of NT, oe21 and *lmd*. (**G**) A branch of *lmd*. (**H**) Result of Evans’ blue staining. We can see few lesions on the leaf before staining. After staining, we can see more stained spots on the leaf. Bars = 50μm. (**I**) Callose deposition. (**J**) Lesions of the *lmd* leaves under a stereomicroscope. The number of lesions increased as leaf aged. Bars = 1 mm.

The GH3 family is an important class of early auxin-response genes involved in the development of the hypocotyls and roots in *Arabidopsis thaliana*. *BpGH3*.5 is a GH3-like gene in birch, which can regulate root elongation as an early auxin-response factor. We obtained a T-DNA insertion mutant named *lmd* when studying the BpGH3.5 function in *Betula platyphylla* × *B*. *pendula*. The *lmd* showed necrotic spots on mature leaves with early leaf senescence. That was different from the other 20 *BpGH3*.5 overexpression lines. The necrotic spots appeared from the edge of the *lmd* leaves. The number of the lesions increased as leaf aged but the lesion size was limited (Fig. 1G and J). Interestingly, we observed the same phenotype on the seedlings growing in woody plant medium (WPM). This indicated that the necrotic lesions were not caused by microorganism but spontaneous. We judged that the *lmd* mutant was a member of lesion mimic mutants. Before this, few lesion mimic mutants in xylophyta were reported. Therefore, we performed a series of experiments to confirm this result.

The *lmd* showed slower growth than its counterparts NT (non-transgenic line) and oe21 (another *BpGH3*.*5* transgenic line) (Fig. 1A,B and F). The leaf net photosynthetic rate was lower in *lmd* (Fig. 1C), which may account for the low increment of *lmd*. To examine the cell death of *lmd*, we performed Evans’ blue staining. Evans’ blue which can enter dead cells is a histochemical indicator of cell death. The staining result showed that *lmd* exhibited a few deep blue spots all over the leaves, even though we could see few necrosis spots on leaves before staining, indicating that necrotic spots would appear subsequently in these positions (Fig. 1H). However, the NT and oe21 leaves did not exhibit positive Evans’ blue staining (Supplemental Fig. S1). To quantify the dead cells in *lmd*, Evans’ blue was eluted by SDS-methanol solution, and the absorbance of the eluent was measured in 595 nm. The absorbance of *lmd* eluent was much higher than that of NT and oe21, which indicated that the dead cells in *lmd* was much more (Fig. 1D). Malondialdehyde (MDA) content indirectly reflects the degree of cellular damage. The MDA content of *lmd* was significantly higher than that of NT and oe21 (Fig. 1E), which indicated that cellular damage occured in *lmd*. The aniline blue staining showed an obviously callose deposition in *lmd* (Fig. 1I).

### Cellular structure observation

**Figure 2:**
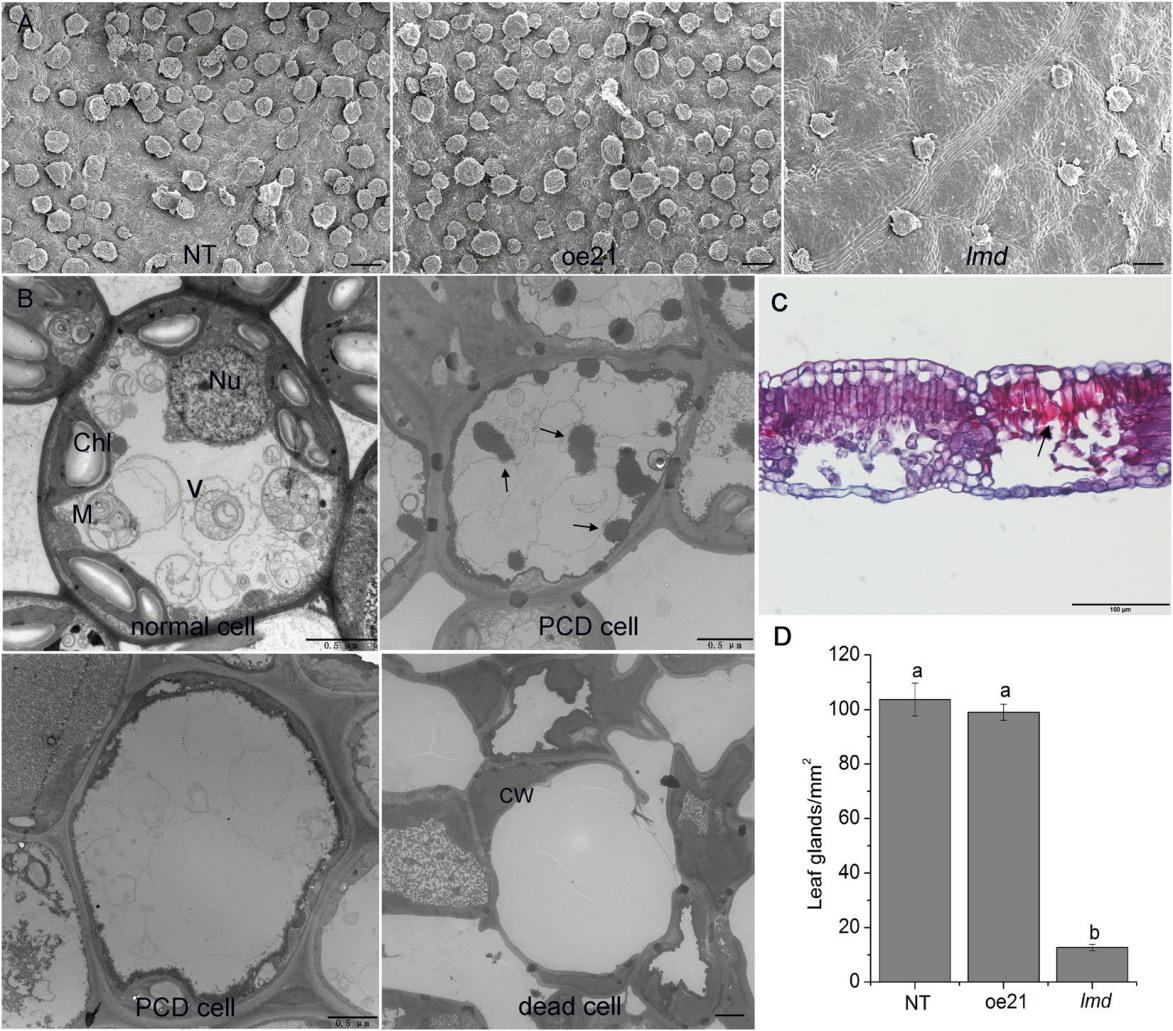
Observation of tissular and cellular structure. (**A**) SEM observation of *lmd*, NT and oe21. Bars = 100 μm. (**B**) TEM observation result showing vacuole (V), nucleus (Nu), chloroplast (Chl), mitochondria (M), cell wall (CW) and autophagosome (the arrows). Bars = 0.5 μm. (**C**) Paraffin section showing cell death was limited in certain area (the arrow). Bar = 100 μm. (**D**) Leaf gland numbers of the 3 lines.

Programmed cell death must be accompanied by cellular structure changes. To observe if there were any cellular structure changes in *lmd*, we performed obversation with both light microscope and electron microscope. Light microscope observation showed that the dead cells clustered in certain areas, but the cells nearby were absolutely normal. It indicated that *lmd* was an initiation type of LMM (Fig. 2C). We observed cell ultrastructure of *lmd* leaves using a transmission electron microscope (TEM). More dead cells were observed in *lmd* compared with oe21 and NT. Some cells generated phagocytosis, and the organelles disappeared, leaving an empty cell wall at last where the lesions were in *lmd* (Fig. 2B). This result was indicative of typical programmed cell death development in plants. There were many leaf glands on the leaf hypodermis of birch, which would degenerated upon leaf maturity. Owing to *lmd* showed early leaf senescence, we scanned the leaf surface using a scanning electron microscope (SEM) to see if there were any changes in *lmd*. On the 3^rd^ leaves, we found fewer leaf glands on *lmd* leaves than on NT and oe21 leaves (Fig. 2A,D). On the 4^th^ leaves, there was no difference among NT, oe21 and *lmd*. The number of leaf glands showed no difference between the 3^rd^ and the 4^th^ leaves in *lmd*. SEM observation showed that the degeneration of leaf glands in *lmd* occurred earlier than in NT and oe21, which was evident from both the number and morphology of the leaf glands. It indicated an early maturity happened in *lmd*.

### Transcriptome sequencing explore transcriptional changes in *lmd*

**Figure 3:**
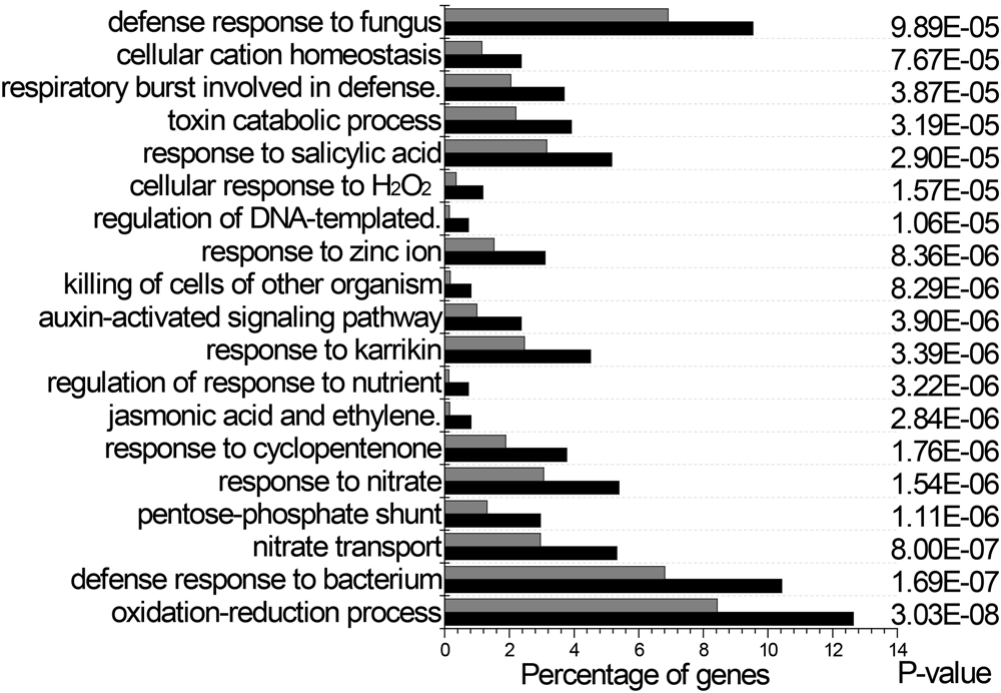
Enriched GO terms of biological process. Grey bars represent the percentage of genes corresponding to each GO term among genes in the cluster, whereas black bars represent the percentage of genes corresponding to these GO terms in the whole birch genome. All of these terms were significantly enriched (P values are indicated to the right of the graphs).

To further learn *lmd* at the molecular level, we used an Illumina 2500 platform to perform *lmd*, oe21 and NT transcriptome sequencing. After filtering the low-quality reads, we obtained 3.46 Gb clean reads of each sample on average. Q30 percentages were all above 91.20%. In total, 23,209,269 (80.40%) of the *lmd*, 27,280,130 (81.95%) of the oe21 and 22,584,972(82.30%) of the NT reads were mapped onto the reference genome. Statistical analysis identified 995 differentially expressed genes (DEGs) among *lmd*, oe21 and NT (Supplemental Fig. S2), of which 560 genes were up-regulated and 435 genes were down-regulated. Many pathogenesis-related genes, such as WRKY, glutathione-S-transferases(GST), ethylene response factor(ERF) and serine/threonine-protein kinase, which have been previously implicated in defence response^39, 40^, were among the significantly up-regulated fraction. Gene Ontology (GO) analysis revealed that DEGs involved in catalytic activity, electron carrier activity, antioxidant activity, immune system process and response to stimulus were enriched (Fig. 3). KEGG analysis indicated that DEGs were enriched in plant hormone signal transduction, plant-pathogen interaction and peroxisome pathways.

### H_2_O_2_ accumulation in *lmd*

**Figure 4:**
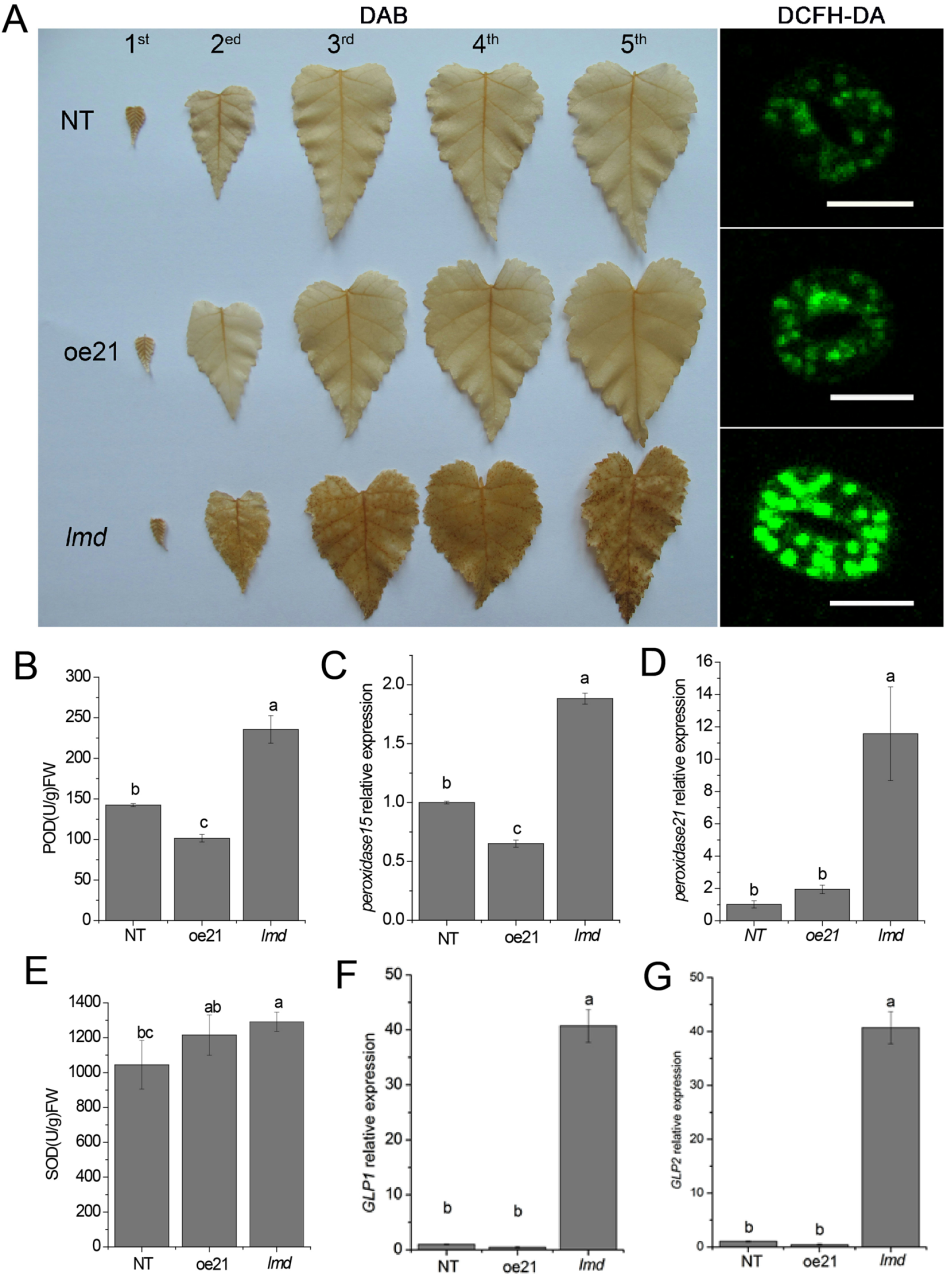
H_2_O_2_ accumulation and activities of POD and SOD in NT, oe21 and *lmd*. (**A**) DAB and DCFH-DA staining for H_2_O_2_ observation. Bars = 20 μm. (**B**) Comparison of POD activity in NT, oe21 and *lmd*. (**C** and **D**) Show the relative expression level of *peroxidase* 15 and *peroxidase* 21. (**E**) Comparison of SOD activity in NT, oe21 and *lmd*. (**F** and **G**) Show the relative expression level of *GLPs*.

The formation of many LMM necrotic lesions is associated with ROS. To determine whether the programmed cell death of *lmd* was accompanied by ROS, we performed 3,3C-diaminobenzidine (DAB) and 2,7-dichlorodihydrofluorescein diacetate (DCFH-DA) staining. DAB staining showed that lots of brown spots occured on the *lmd* leaves (from 1^st^ to 5^th^ leaves, including immature and mature leaves), whereas the leaves of oe21 and NT showed a negative reaction. It indicated H_2_O_2_ accumulated in *lmd* (Fig. 4A). DCFH-DA can move freely through the cytomembrane. It indicates the level of intracellular ROS. DCFH-DA staining showed a higher accumulation of ROS in *lmd* (Fig. 4A). Peroxidases (PODs) are involved in plant resistance to biotic and abiotic stresses. The POD activity of *lmd* was higher than that of NT and oe21 (Fig. 4B). Superoxide dismutase (SOD) is a primary biological scavenger of free radicals. It is an important antioxidant enzyme. SOD activity in the *lmd* mutant was higher than that of NT but was not significantly different from that of oe21 (Fig. 4E). To confirm the results, we performed qRT-PCR to measure the relative expression level of peroxidase genes (*peroxidase* 15, *peroxidase* 21) and *GLPs* (*GLP1 and GLP2*). Our results indicated higher relative expression level of *peroxidase* 15, *peroxidase* 21 and *GLPs* in *lmd* than in NT and oe21 (Fig. 4C,D,F and G). All above indicated that H_2_O_2_ accumulated in *lmd* and the activities of enzymes related to ROS were increased.

### SA involvement in enhanced resistance of *lmd*

**Figure 5:**
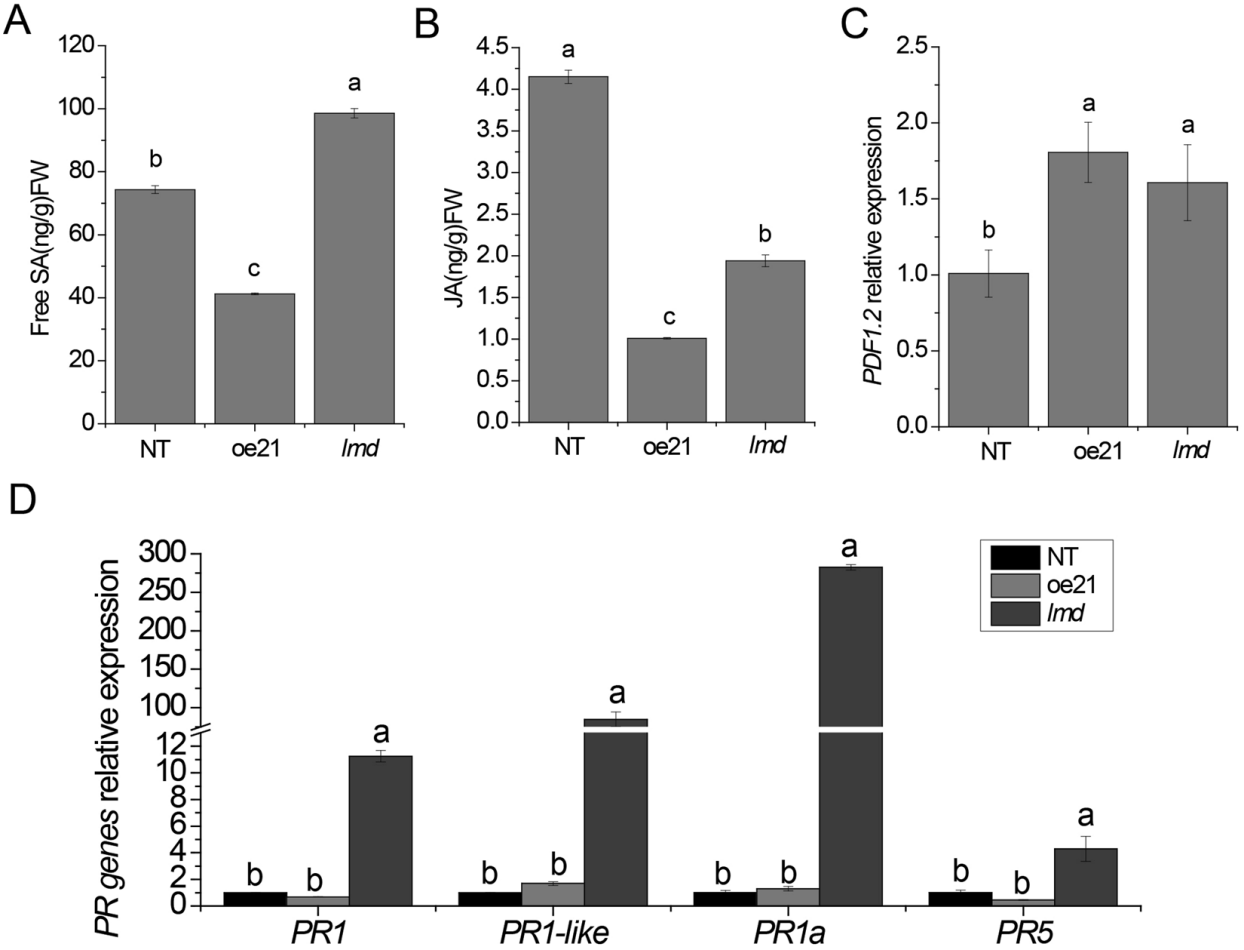
Phytohormones measurements and qRT-PCR results of *PRs*. (**A**) Free SA content. (**B**) JA content. (**C**) *PDF1*.2 relative expression level. (**D**) *PR genes* relative expression level. *PR* genes relative expression level in *lmd* were all significantly more than those of NT and oe21.

**Figure 6:**
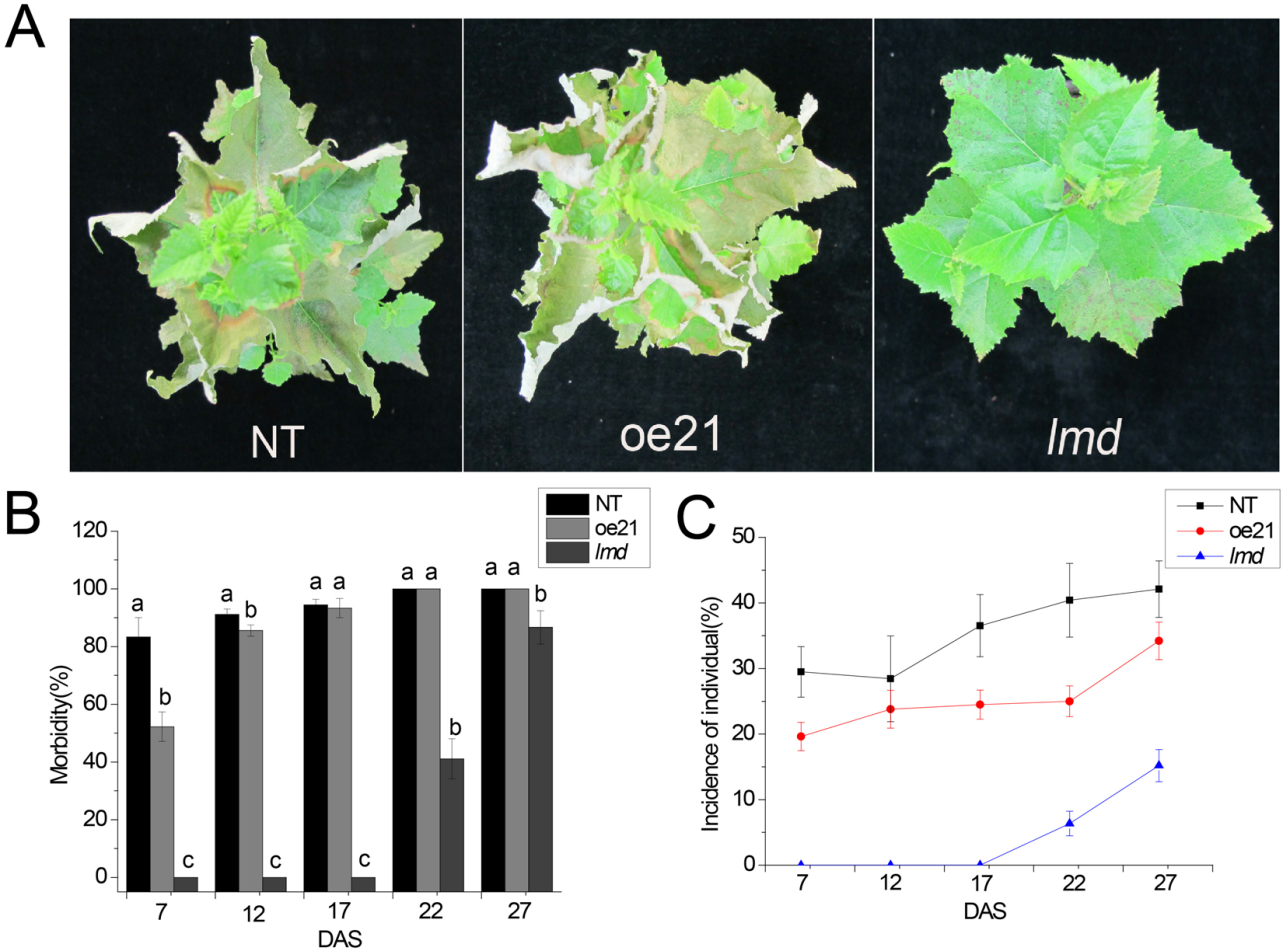
*lmd* is more resistant to *Alternaria alternate*. (**A**) Phenotype of NT, oe21 and *lmd* 12 days after spraying (DAS) with *Alternaria alternate* spores. (**B**) Morbidity of NT, oe21, and *lmd* observed at 7, 12, 17, 22, and 27 days after spraying. (**C**) Individual incidence of NT, oe21 and *lmd* at 7, 12, 17, 22, and 27 days after spraying.

Disease resistance is regulated by multiple signal transduction pathways such as SA, JA and ET^41^. We performed gas chromatography to measure the ethylene release. The ethylene release of *lmd*, NT and oe21 showed no difference at 1 ppm. HPLC-MS assay for measuring SA and JA content showed that SA accumulated in *lmd* (Fig. 5A). The JA content in *lmd* was lower than that of NT but higher than that of oe21 (Fig. 5B). There are some molecular markers for each pathway. SA usually induces the expression of *PRs* to enhance disease resistance in plants^42^. PDF1.2 is a marker for the ET/JA pathway. Therefore, we performed qRT-PCR to quantify these genes. *lmd* showed a high expression level of *PRs*, including *PR1*, *PR1-like*, *PR1a* and *PR5* (Fig. 6D). *PDF1*.2 expression level showed no difference compared with oe21 (Fig. 5C). All of the above results indicated that SA accumulated and the expression of *PRs* was induced in *lmd*.

### Enhanced resistance to *Alternaria alternata*

Many LMM exhibite enhanced resistance to pathogens^43, 44^. To test whether the *lmd* mutant was resistant to *Alternaria alternata*, we sprayed fresh spore suspension onto NT, oe21 and *lmd* seedlings. 7 days later, NT and oe21 were heavily infected with several wilted and flag leaves, whereas few wilted leaves were observed in the *lmd* mutant, despite the many lesions on its leaves (Fig. 6A). 17 days after spraying (DAS), the morbidity of both NT and oe21 were above 90%, but still no *lmd* plants were infected. The onset time of *lmd* was delayed for 15 days at least, which was 22 days after spraying. At that time, the morbidity of *lmd* was 45.55%.The morbidities of NT and oe21 were both upto 100% (Fig. 6B). To measure the disease severity, we counted the ratio of infected leaves to all leaves on one plant (named individual incidence). We can see the the ratio of infected leaves on each plant in *lmd* was much lower than that of NT and oe21 (Fig. 6C). It indicated *lmd’s* resistance to *Alternaria alternata* was enhanced.

## Discussion

We obtained 21 *BpGH3*.5 overexpression transgenic lines when studying the function of BpGH3.5 in 2012. One of them showed necrotic spots on the mature leaves. The number of the necrotic spots increased as leaf aged. The leaves of this line dropped earlier than the other 20 transgenic lines. Molecular and histochemical assays indicated that this transgenic line was a lesion mimic mutant. We named it *lmd* (lesion mimic and early deciduous leaf). Lesion mimic mutants have been reported in many species^45, 46^, but little was reported in xylophyta. The *lmd* mutant showed H_2_O_2_ accumulation from the 2^nd^ leaves to the 5^th^ leaves (the 1^st^ leaves were too small to observe). H_2_O_2_ is reported as a cell death inducer. We examined cell death using Evans’ blue staining. Our result indicated that there was little cell death in the 2^nd^ leaves of *lmd* except for few spots at the edge of the leaves. The cell death increased in the 3^rd^ and 4^th^ leaves in *lmd*. The H_2_O_2_ accumulated in the 2^nd^ leaves, but the cell death was little. It indicated that cell death induced by H_2_O_2_ was related to the development of leaves in *lmd*. Plants can form protective physical barriers, such as callose, to protect themselves from invasion. Callose is a multifaceted defence response that depends on the environmental conditions and the challenging pathogen-associated molecular patterns^47^. Aniline blue staining showed callose deposition in *lmd* leaves. qRT-PCR showed that the relative expression level of β-1,3-glucanase (callose degradation)^48^ gene was lower in *lmd*, which indicated a transcriptional change of related genes (Supplemental Fig. S3).

LMM can be divided into two types: initiation mutants and propagation mutants^49^. Microscope observation showed the dead cells were limitied in certain areas and the other areas showed absolutely normal. This result was in line with the Evans’ blue staining result. We can conclude that *lmd* is an initiation class of LMM. Plant PCD is different from animal PCD. Basing on rupture of the tonoplast followed by rapid clearance of the whole cytoplasm and sometimes most of the cell walls or not, plant PCD can be grouped into two types: autolytic and non-autolytic^50^. Ultrastructure observation indicated that many cells generated autophagosomes in *lmd*, but few autophagosomes was found in NT and oe21 cells. Dead cells clustered in *lmd*, leaving only the cell wall. That’s different from NT and oe21.

Transcriptome analysis explored the gene interactions and the change of transcriptional level in *lmd* leaves. WRKY superfamily transcription factors (TFs) play an important role in plant growth, development and responses to biotic and abiotic stress^39, 51^. *NPR1 i*s a key regulator of the SAR pathway downstream of SA. In *Arabidopsis*, WRKY TFs act upstream or downstream of NPR1 to mediate defence responses to the pathogen^52^. Many WRKY TFs such as WRKY9, 19, 28, 32, 33, 37, 70, 71, 72, and 75 were all up-regulated which indicated defence responses and the regulation of plant growth changed in *lmd*. Some other TFs or proteins associated with defence response, senescence, hormone signals, and oxidation-reduction are also up-regulated. This indicated a complex signal network formation in *lmd*.

Some environmental factors such as light^53, 54^, temperature, and humidity^55, 56^ can influence the phenotype of LMM^57^. Both light and temperature influence the chloroplast number and morphology in the FZL mutant^58^. Certain mutants show total or partial degradation of chloroplast membranes and irregular degradation of thylakoids. In this study, we cultured the *lmd* mutant under natural conditions (45°43.2′N, 126°37.4′E). During the whole growing season (from May to October), plants experience different sunshine condition (long-day and short-day) and different temperatures (5 °C to 34 °C). The *lmd* mutant including one-year-old to five-year-old plants, showed necrotic spots on mature leaves steadily. We also simulated long-day (16 h light) and short-day (8 h light) conditions, and the necrotic spots on *lmd* leaves showed no difference. Temperature treatments of 30 °C and 16 °C did not result in changes in the formation of lesions. That meant the necrotic spots formation of *lmd* was independent of light and tempreture.

Many studies showed that chloroplasts play an important role in the formation of lesions^7, 58^. We tested the photosynthesis and chlorophyll fluorescence of *lmd*, oe21 and NT. The net photosynthetic rate (Pn) of *lmd* was lower than that of NT and oe21. The maximum PSII efficiency (Fv/Fm), photochemical efficiency of PSII (ΦPSII), photochemical quenching (qp), non-photochemical quenching (NPQ), stomatal conductance, intercellular CO_2_ concentration and transpiration rate in *lmd* showed no differences with oe21 and NT (Supplemental Table S1). The lower Pn may be due to the lesions on leaves. Ultrastructure observation showed that the number and morphology of chloroplasts in *lmd* was not different from those of oe21 and NT. These results indicated that the formation of lesions was not related to chloroplast in *lmd*.

Ethylene is considered as an important phytohormone related to plant senescence and fruit ripening^59^. Some studies also showed ethylene can promote leaf senescence at a defined age, but it is not the most significant factor^60^. We examined the ethylene release of *lmd* by gas chromatography. Our results (measuring at 1 ppm) showed that there was no difference among *lmd*, NT and oe21 on ethylene release which indicated that the early drop of leaf in *lmd* was independent of ethylene. Ethylene is not the most important factor for leaf dropping in *lmd*. In addition to ethylene, abscisic acid (ABA) is another important plant hormone for leaf abscission. HPLC-MS showed that ABA of *lmd* was higher than that of oe21 and NT, which may account for the early drop of leaves in *lmd* (Supplemental Fig. S4).

**Figure 7:**
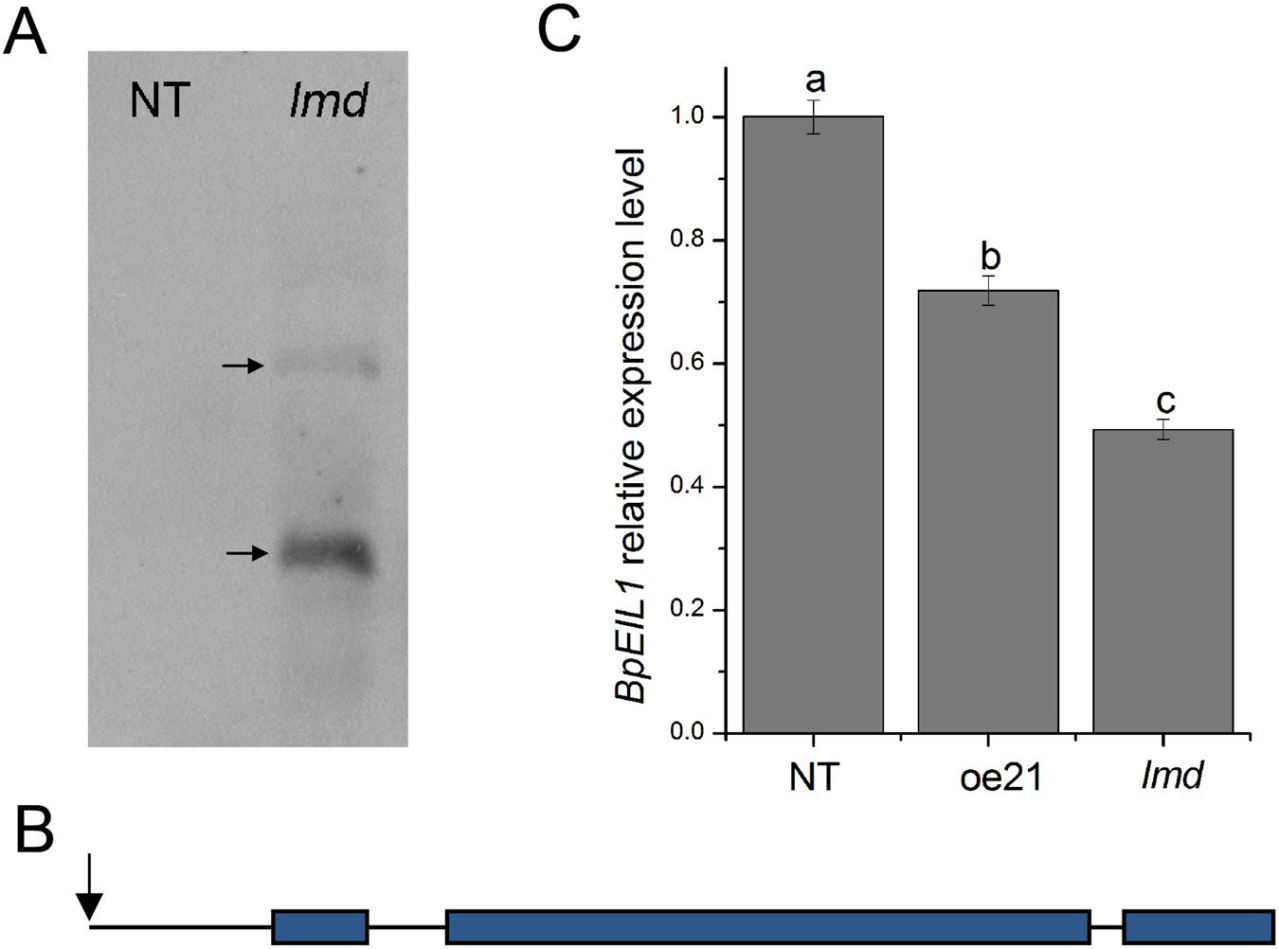
The T-DNA insertion sites analysis. (**A**) Southern blotting result. The arrows indicate that there are two T-DNA insertion sites in *lmd* genome. (**B**) Structure of *BpEIL1* gene. Boxes indicate exons, lines indicate introns, the arrow indicates the insertion site of T-DNA which is 598 bp before the first exon of *BpEIL1*. (**C**) *BpEIL1* relative expression level of NT, oe21 and *lmd* determined by qRT-PCR. The expression level of *BpEIL1* was significantly lower than that of NT and oe21.

BpGH3.5 is an early auxin-response factor which regulates root elongation in birch^61^. Since the other 20 *BpGH3*.5 overexpression lines do not show a phenotype similar to *lmd*, the phenotype of *lmd* was not a result of the overexpression of *BpGH3*.5 but was related to the T-DNA insertion. Through the southern blotting assay, we found two insertion sites in *lmd* genome (Fig. 7A). Thus, we performed assays to confirm the T-DNA insertion sites. Genome re-sequencing is mainly used to analyse differences between individuals in a species and also a powerful tool to study plant or animal evolution^62–64^. In this study, we used it to confirm the T-DNA insertion sites and achieved a fine result in line with the TAIL-PCR result (Supplemental Fig. S5). Using the genomewide re-sequencing approach, a total of 56,361,128 clean reads were generated, covering 91.97% of the reference Betula genome. We used the L-border and R-border sequences of T-DNA to blast the re-sequencing data and then used the output reads to blast the Betula genome data. According to the blast results (Table 1), we achieved two insertion positions: one was a non-gene-coding region and the other was 598 bp before the first exon of *BpEIL1* (Fig. 7B). qRT-PCR showed that the *BpEIL1* expression level of *lmd* was significantly lower than that of NT and oe21 which indicated that the insert inhibited the expression of *BpEIL1* (Fig. 7C). EIL1 is a member of the EIN3 family, which is an important transcription factor in ethylene signal transduction and junction of ET and other phytohormones^65^. In *Arabidopsis*, EIN3 and EIL1 are functional redundant. There are only two members of EIN3 family in birch which indicated that *BpEIN3* and *BpEIL1* may have their own special functions respectively. In *Arabidopsis*, through the negative regulation of SID2, EIN3/EIL1 have an inhibiting effect on SAR^34^. In rice spotted leaf3 (SPL3) mutant, *EIN2* and *EIN3* are both down-regulated^66^. But there are no reports to show that EIN3/EIL1 can directly induce lesion mimic phenotype in other species. However, gene functions may differ in different genetic backgrounds^58, 67^. In this study, the lower *BpEIL1* expression level in *lmd* may result in a lesion mimic phenotype through H_2_O_2_ accumulation and SA increasing. However, how BpEIL1 regulates the lesion mimic phenotype formation still needs to be discussed.

**Table 1:** Results of genome re-sequencing in *lmd*.

Reference genome ID	Locations	Reads number	Comparing results
scaffold1651_cov0	116149–120471	96	*BpGH3*.5
scaffold864_cov0	1197731–1198827	26	598 bp before *BpEIL1*
scaffold864_cov0	1224752–1225831	4	Intergenic region

## Material and Methods

### Plant material

The *lmd* mutant was isolated from the *BpGH3*.5 transgenic lines. In all experiments, we used both the *BpGH3*.5 overexpression lines *lmd* and oe21 and the non-transgenic line NT. All the plants used in this study were 3 years old unless otherwise mentioned. Plants were grown under the natural conditions of Harbin in Heilongjiang province of China. Some plants were grown in the culture room with 16 h/8 h light/dark at a temperature of 26 °C.

### Plant height and ground diameter measurement

Plant height was measured using a metre stick. The ground diameter was measured using Vernier callipers. Thirty plants from each line were measured.

### Callose deposition

The prepared paraffin sections were dewaxed with 100% xylol twice and then dehydrated in an ethanol series of different concentrations. After being rinsed by distilled water, the samples were dipped into 0.01% aniline blue for 1 h. Afte rinsing with glycerine, the sections were examined under a ZEISS AXIO Imager. A1 fluorescence microscope.

### Chlorophyll Fluorescence and Photosynthesis Measurement

The functional leaves from the upper branch of triennial plants were used as materials. Chlorophyll fluorescence was measured using PAM-2500. Photosynthesis was measured using LI-6400XT. Ten plants from each line were measured.

### Detection of cell death

The functional leaves were harvested and placed in 0.25% Evans’ blue solution for 30 min. The chlorophyll was removed by soaking in 95% ethanol until the tissues were free of chlorophyll. For measuring the dead cells, we eluted Evans’ blue by boiling 100 mg of stained leaves in 2 mL of SDS-methanol (methanol 50%: SDS 1%) solution at 50 °C for 1 h. After filtered, the eluent was measured using a 722 s spectrophotometer at 595 nm. The assay was performed in triplicate.

### Cellular structure observation

Functional leaves from *lmd*, oe21 and NT were used as experimental materials. For microscopic observation, the leaves were cut into tablets (5 mm × 10 mm) and dipped into FAA for 24 h and then embedded in paraffin. The paraffin sections were stained by safranin-fast green. The leaves were collected and pre-fixed with a mixed solution of 2.5% glutaraldehyde for TEM and SEM observation. For TEM, the samples were post-fixed in 1% osmium tetroxide, dehydrated in an acetone concentration series, infiltrated in epoxy for 3 h and then embedded. The prepared tissue was cut with a diamond knife, stained with uranyl acetate and lead citrate, and then observed under a JEM-100X transmission electron microscope^68^. For SEM, we took the 1^st^ to the 4^th^ leaves as metarials. The samples were dehydrated in an ethanol concentration series and tertiary butanol and dried in an ES-2030 (HITACHI) freeze-drier for 4 h. The prepared tissue was fixed on a sample platform, coated with 100–150 Å gold, and observed under an S-3400 scanning electron microscope.

### DAB staining

For the detection of H_2_O_2_ accumulation, fresh leaves from *lmd*, oe21 and NT were stained with 1 mg/ml (pH 3.8) DAB overnight at room temperature, followed by soaking in 95% ethanol until the chlorophyll was eluted.

### DCFH-DA staining

Dimethyl sulfoxide (DMSO) was used to dissolve DCFH-DA to 10 μM. The functional leaves were torn from the hypodermis and immersed into the stomatal opening solution (30 mM KCl, 10 mM MES-KOH, pH 6.15) to induce stomatal opening under light. This was then placed into the DCFH-DA solution and stained for 10–15 min and observed under an LSM 700 laser scanning microscope.

### RNA-seq libraries construction, sequencing, functional annotation and GO analysis

RNA-seq libraries were constructed from mRNA using NEB Next UltraTM RNA Library Prep Kit for Illumina (NEB, USA) according to the manufacturer’s instructions and sequenced on an Illumina Hiseq. 2500 platform. Gene function was annotated based on the following databases: Nr (NCBI non-redundant protein sequences), Nt (NCBI non-redundant nucleotide sequences), Pfam (Protein family), KOG/COG (Clusters of Orthologous Groups of proteins), Swiss-Prot (A manually annotated and reviewed protein sequence database), KO (KEGG Ortholog database), GO (Gene Ontology). GO enrichment analysis of the differentially expressed genes (DEGs) was implemented by the GOseq R packages based on Wallenius non-central hyper-geometric distribution^69^.

### RNA extract and quantitative real-time PCR

Total RNA was extracted from the leaves of *lmd*, oe21 and NT using a Plant RNA Extract kit (Bioteke, Beijing, China). cDNA was synthesized using 1 μg of total RNA by ReverTra Ace qPCR RT Master Mix with gDNA Remover (TOYOBO, OSAKA, Japan) according to the manufacturer’s instructions. Quantitative real-time PCR (qRT–PCR) was performed on an ABI 7500 real-time PCR detection system using SYBR Green Real-time PCR Master Mix -Plus- (TOYOBO, OSAKA, Japan). *Bp18S* was chosen as a reference gene to normalize the relative expression. Gene-specific primers (Supplemental Table S2) were used to amplify each gene in triplicate.

### Phytohormone and enzyme assays

Phytohormones were extracted from plant leaves with isopropanol/hydrochloric acid and dichloromethane and then dried using nitrogen. The residue was dissolved in methanol. After filtered by a 0.22-µm filter membrane, the phytohormones content was measured by HPLC-MS. The ethylene release was measured by gas chromatography as reported^70^. The total soluble protein content, MDA content, SOD activity, and POD activity of the leaves were determined using a total protein assay kit (A045-3), a thiobarbituric acid (TBA) assay kit (A003-1), an SOD assay kit (A001-1), and a POD assay kit (A084-3), respectively, all of which were acquired from the Nanjing Jiancheng Technology Company (Nanjing, China)^68^. All of the physiological and biochemical indexes above were measured in triplicate.

### Pathogen injection

*Alternaria alternata* was cultured on potato dextrose agar (PDA) medium at 25 °C for 7 d. The spores were suspended in sterile water to an density of 2 × 10^5^/mL and then sprayed onto 40-cm-high seedlings. The treated seedlings were cultured at 25 °C and 16 h/8 h light/dark with 1000–1500 lx intensity. 7 days later, we started to record the disease occurrence. Mobility was calculated by the rate of infected individuals. The severity of the infected plants was calculated by the proportion of infected leaves relative to all leaves on each plant. The assay was performed for 3 times.

### Southern blotting and TAIL-PCR

DNA was extracted from the loose callus of *lmd* using the cetyl trimethylammonium bromide (CTAB) method^71^. Southern blotting was performed as reported^72^. Briefly, total DNA was digested by BamH I and separated by gel electrophoresis. Molecules were transferred from the gel to a nylon membrane by capillary action using absorbent paper. A specific sequence was detected on the membrane by molecular hybridization with DIG-labelled 35 S promotor fragment as probe (Supplemental Fig. S6). CDP-star (Roche, Mannheim, Germany) was used to detect the signal. TAIL-PCR was performed following TaKaRa Genome Walking Kit (TaKaRa, Daliang, China) instructions. Three gene-specific primers (Supplemental Table S2) were used in this assay.

### DNA isolation and re-sequencing

Genomic DNA of *lmd* leaves were isolated using the CTAB method^71^. Illumina sequencing library was constructed according to manufacturer’s instructions and then sequenced on the Illumina Hiseq. 2500 platform. The Betula genome was download from http://birch.genomics.cn/ as reference genome. We searched for the reads containing L-border or R-border sequence of T-DNA in the clean reads data. The searching results were used to blast the reference genome to look for the insertion sites.

### Data analysis

The treatments were analysed for significant differences using one-way ANOVA. Data are presented as the mean ± standard error.

## Electronic supplementary material

supplemental information
