# Supplementary material for: Characterization and Identification of a woody lesion mimic mutant lmd, showing defence response and resistance to Alternaria alternate in birch
Source: Sci Rep. 2017 Sep 12;7:11308. doi: 10.1038/s41598-017-11748-2 (PMC5595973; doi:10.1038/s41598-017-11748-2)
Supplement: Supplementary file 1 — supplemental information [file 41598_2017_11748_MOESM1_ESM.pdf]

Characterization and Identification of a woody lesion mimic mutant *lmd*, showing defence response and resistance to *Alternaria alternate* in birch

Ranhong Li[1,2], Su Chen[1], Guifeng Liu[1], Rui Han[1], Jing Jiang[1]*

1 State Key Laboratory of Tree Genetics and Breeding, Northeast Forestry University, Harbin 150040, China

2 Mudanjiang Normal University, Mudanjiang 157100, China

*Correspondence: jiangjing196010@126.com Telephone: 13946026246

Supplemental Table S1 Data related to Photosynthesis and Chlorophyll Fluorescence

	Cond	Ci	Trmmol	Fv/Fm	$\Phi_{PS\,II}$	qp	NPQ
NT	0.268±0.042	284.03±13.537	6.033±0.610	0.772±0.004	0.645±0.021	0.898±0.015	0.326±0.066
oe21	0.183±0.047	247.8±20.038	4.692±0.650	0.788±0.003	0.668±0.021	0.894±0.018	0.260±0.070
lmd	0.171±0.034	276.556±10.685	4.423±0.556	0.783±0.008	0.688±0.005	0.919±0.016	0.210±0.007

Supplemental Table S2 Primers used in this work

Primers	Sequence(5'--3')
PR1-F	ACTCATGTGTTGGCGGGGAA
PR1-R	GTTGCCTGGAGGGTCGTAGT
PR1-like-F	TGCCCAAGACACCCAACAAGA
PR1-like-R	CGTTCACCCACAAGTTCACCG
PR1a-F	CGAACAGCCCTTACGGTGAA
PR1a-R	CCAGCAGCGCAAGAGTTAGA
PR5-F	TGGCCAGGAACTCTAACATCGG
PR5-R	CGTTCGTGGTGCATCGTGTT
peroxidase15-F	TTTACGCTAGCACATGCCCG
peroxidase15-R	GAAGGCGGATGAGTTTGGCA
peroxidase21-F	CGTTCTATCAGGAGCGCACA
peroxidase21-R	TAGGAGGGACGAATCCAGCG
PDF1.2-F	AAGAGATGGTGAGGCCAAGTGAG
PDF1.2-R	TTCTGCACACCAGAGCACAGTT
BpEIL1-F	GAATGAACCTCACATTAGGCCAGAG
BpEIL1-R	CCTCCACAATCTAATGACAGGCTATC
GLP1-F	CATTTCCGGGGCTCAACACT
GLP1-R	CTGAAGCGACCAGAACAGCC
GLP2-F	GTTTTGACAGCTGGACAGCTTTTTATC
GLP2-R	CGAAAGCAATCCCTGGTAATTGACT
CalS-F	AAGGGGCTTCCGTAAAGCGT
CalS-R	ACCTCTGTATCGTGCACCCC

BG-F	TCGAACTTGGCAACGAACGC
BG-R	TTCCCAACAAGGATGCCGGT
SP1	TCGCCAGTCTTTACGGCGAGTTCTG
SP2	GCAAGCCTTGAATCGTCCATACTGG
SP3	ACCTGCTGCGTAAGCCTCTCTAACC

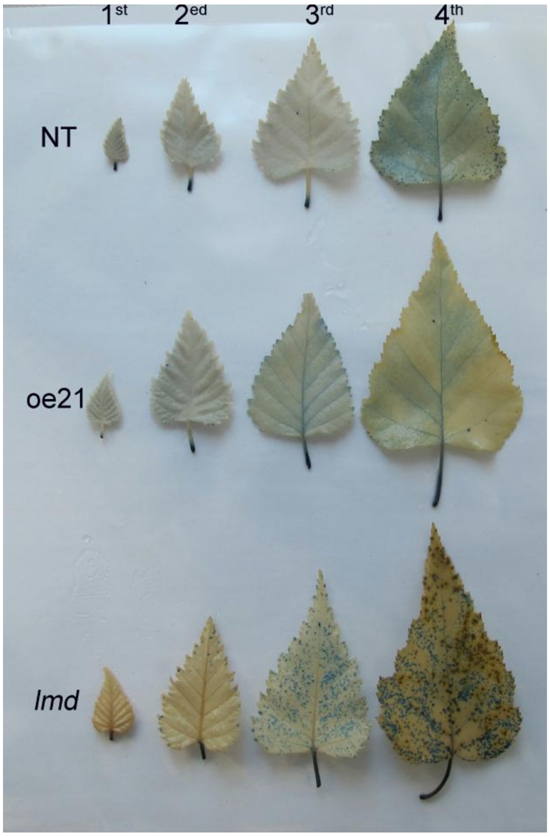

Supplemental Fig. S1 Evans' Blue Staining of NT, oe21 and *lmd* (from the 1st to the 4th leaves)

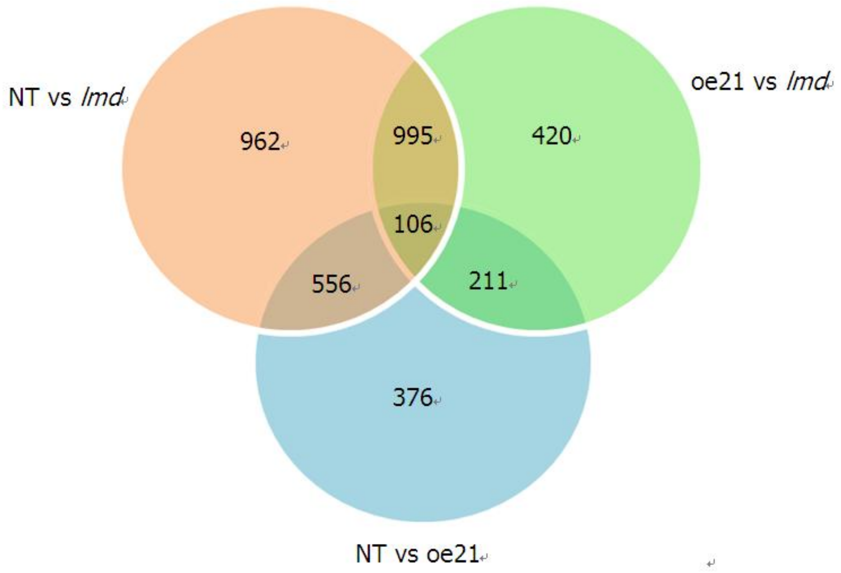

Supplemental Fig. S2 Venn diagram of DEGs among NT, oe21 and *lmd*

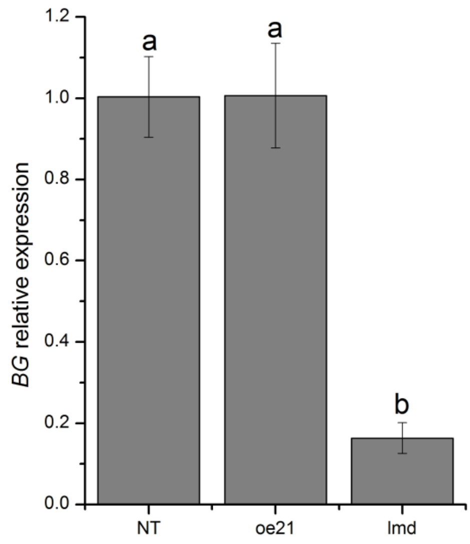

Supplemental Fig. S3 qRT-PCR analysis of β-1,3-glucanase(*BG*) transcriptional level

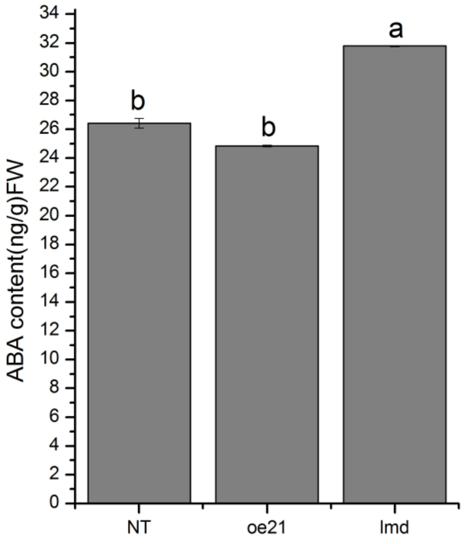

Supplemental Fig. S4 ABA level of NT, oe21 and *lmd*

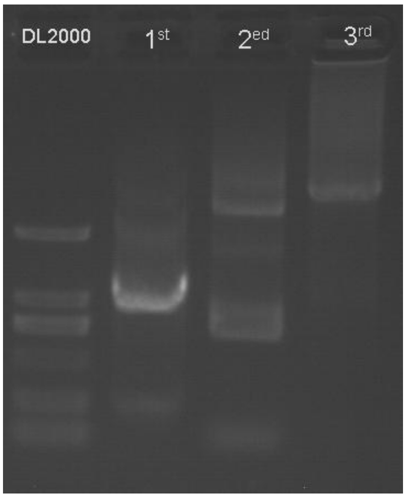

Supplemental Fig. S5 TAIL-PCR Analysis of *lmd*

TACGCAGCAGGTCTCATCAAGACGATCTACCCGAGCAATAATCTCCAGGAAATCAAAT
ACCTTCCCAAGAAGGTTAAAGATGCAGTCAAAAGATTCAGGACTAACTGCATCAAGA
ACACAGAGAAAGATATATTTCTCAAGATCAGAAGTACTATTCCAGTATGGACGATTCAA
GGCTTGCTTCACAAACCAAGGCAAGTAATAGAGATTGGAGTCTCTAAAAAGGTAGTT
CCCACTGAATCAAAGGCCATGGAGTCAAAGATTCAAATAGAGGACCTAACAGAACTC
GCCGTAAAGACTGGCGAACAGTTCATACAGAGTCTCTTACGACTCAATGACAAGAAG
AAAATCTTCGTCAACATGGTGGAGCACGACACACTTGTCTACTCCAAAAATATCAAAG
ATACAGTCTCAGAAGACCAAAGGGC

Supplemental Fig. S6 Probe sequence used in Southern-blotting
